# Supplementary material for: Identification of photoperiod-induced specific miRNAs in the adrenal glands of Sunite sheep (Ovis aries)
Source: Front Vet Sci. 2022 Jul 22;9:888207. doi: 10.3389/fvets.2022.888207 (PMC9354845; doi:10.3389/fvets.2022.888207)
Supplement: Supplementary file 1 [file Data_Sheet_1.ZIP › Supplementary file/miRDeep2 file.zip!/miRDeep2 file/Know_Structure/SPLP42A1/oar-mir-26a.pdf]

miRBase precursor : oar-mir-26a
Total read count : 1281894
oar-miR-26a read count : 1281786
remaining reads : 108

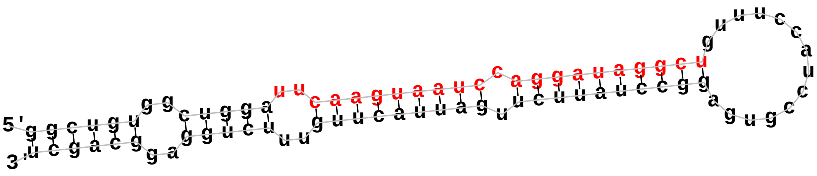

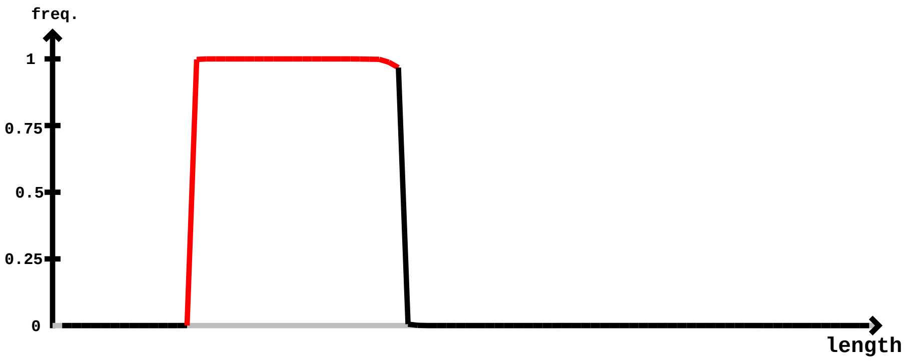

oar-miR-26a

```
5'- ggcuguggcuggauucaaguaauccaggauaggcuguuuccauccgugaggccuauucuugauuacuuguuucuggaggcagcu   -3'    exp
    ((((((..(((((..(((((((((.((((((((((..............))))))))))).)))))))))..)))))..))))))   reads      mm         sample
    ............auucaaguaauccaggaua.....................................................   1          0          seq
    ............Guucaaguaauccaggauag....................................................   1          1          seq
    ............auucaaguaauccaggauagg...................................................   1          0          seq
    ............auucaaguaauccaggauaggc..................................................   8          0          seq
    ............Guucaaguaauccaggauaggc..................................................   2          1          seq
    ............auucaaguaauccaggauaggcu.................................................   212        0          seq
    ............Guucaaguaauccaggauaggcu.................................................   36         1          seq
    ............auucaaguaauccaggauaggcug................................................   1          0          seq
    ............auucaaguaauccaggauaggcugu...............................................   1          0          seq
    .............uucaaguaauccaggau......................................................   526        0          seq
    .............uucaaguaauccagAau......................................................   2          1          seq
    .............uucaUguaauccaggau......................................................   2          1          seq
    .............uucaGguaauccaggau......................................................   1          1          seq
    .............uAcaaguaauccaggau......................................................   2          1          seq
    .............uucaaguaauccaggaA......................................................   21         1          seq
    .............uucaaguaauccaggCu......................................................   1          1          seq
    .............uucaaguaauccaggaG......................................................   22         1          seq
    .............uucaaAuaauccaggau......................................................   1          1          seq
    .............uucaaguaauccaggaua.....................................................   669        0          seq
    .............uucaGguaauccaggaua.....................................................   3          1          seq
    .............uucaaAuaauccaggaua.....................................................   1          1          seq
    .............uucaaguaauccaggauaC....................................................   2          1          seq
    .............uucaaguaauccaggauag....................................................   759        0          seq
    .............uucaaguaauccaggauagg...................................................   13876      0          seq
    .............uucaaguaauccaggauaggc..................................................   25845      0          seq
    .............uucaaguaauccaggauaggcu.................................................   1230463    0          seq
    .............uucaaguaauccaggauaggcug................................................   5623       0          seq
    .............uucaaguaauccaggauaggcugu...............................................   1133       0          seq
    .............uucaaguaauccaggauaggcuguu..............................................   19         0          seq
    .............uucaaguaauccaggauaggcuguG..............................................   5          1          seq
    .............uucaaguaauccaggauaggcuguuu.............................................   7          0          seq
    .............uucaaguaauccaggauaggcuguuuc............................................   1          0          seq
    ..............ucaaguaauccaggauagg...................................................   15         0          seq
    ..............ucaaguaauccaggauaggc..................................................   55         0          seq
    ..............ucaaguaauccaggauaggcu.................................................   2309       0          seq
```

oar-miR-26a

```
ggcuguggcuggauucaaguaauccaggauaggcuguuuccauccgugaggccuauucuugauuacuuguuucuggaggcagcu
..............ucaaguaauccaggauaggcug................................................ 21 0 seq
..............ucaaguaauccaggauaggcugu............................................... 8 0 seq
..............ucaaguaauccaggauaggcuguu.............................................. 1 0 seq
..............ucaaguaauccaggauaggcuguuuc............................................ 1 0 seq
...............caaguaauccaggauagg................................................... 2 0 seq
...............caaguaauccaggauaggc.................................................. 1 0 seq
...............caaguaauccaggauaggcu................................................. 103 0 seq
................aaguaauccaggauagg................................................... 2 0 seq
................aaguaauccaggauaggcu................................................. 18 0 seq
................aaguaauccaggauaggcug................................................ 1 0 seq
..................guaauccaggauaggcu................................................. 2 0 seq
................................................aggccuauucuugauuacuugu.............. 2 0 seq
................................................aggccuauucuugauuacuuguu............. 3 0 seq
................................................aggccuauucuugauuacuuguuu............ 1 0 seq
...................................................ccuauucuugauuacuugu.............. 2 0 seq
...................................................ccuauucuugauuacuuguu............. 5 0 seq
...................................................ccuauucuugauuacuuguuu............ 40 0 seq
...................................................ccuauucuugauuacuuguuuc........... 49 0 seq
...................................................ccuauucuugauuacuuguuucu.......... 3 0 seq
....................................................cuauucuugauuacuuguuu............ 1 0 seq
....................................................cuauucuugauuacuuguuuc........... 2 0 seq
```
